# Association between Blood Manganese Levels and Visceral Adipose Tissue in the United States: A Population-Based Study

**DOI:** 10.3390/nu14224770

**Published:** 2022-11-11

**Authors:** Chengzhe Tao, Yuna Huang, Xuzhao Huang, Zhi Li, Yun Fan, Yan Zhang, Tingya Wan, Liyu Lu, Qiaoqiao Xu, Wei Wu, Di Wu, Feng Zhang, Chuncheng Lu

**Affiliations:** 1 State Key Laboratory of Reproductive Medicine, Center for Global Health, School of Public Health, Nanjing Medical University, Nanjing 211166, China; 2 Key Laboratory of Modern Toxicology of Ministry of Education, School of Public Health, Nanjing Medical University, Nanjing 211166, China; 3 Department of Microbes and Infection, School of Public Health, Nanjing Medical University, Nanjing 211166, China; 4 Obstetrics and Gynecology Hospital, Institute of Reproduction and Development, Fudan University, Shanghai 200011, China

**Keywords:** manganese, visceral adipose tissue, visceral obesity, NHANES, epidemiology

## Abstract

Background: Manganese (Mn) is an essential trace element with a narrow toxic margin for human health. The association between Mn exposure and adverse visceral adipose tissue (VAT) accumulation is unclear. Objective: This study aimed to estimate the associations of blood Mn levels with VAT mass or visceral obesity in the general population in the United States. Method: This cross-sectional study included data of 7297 individuals released by National Health and Nutrition Examination Survey (NHANES). VAT was quantified with dual-energy X-ray absorptiometry, and blood Mn was measured using inductively coupled plasma mass spectrometry. The generalized linear model and generalized additive model (GAM) were applied to estimate the linear and non-linear associations between Mn levels and VAT mass, respectively. Logistic regression was used to estimate the associations between blood Mn levels and the risk of visceral obesity. Results: Fully adjusted generalized linear regression revealed that individuals in the higher quantile of Mn had increased VAT mass compared with those in the lower quantile (β per quantile change = 0.025; 95% CI of 0.017, 0.033; *p* < 0.001). Positive associations were also observed in males and females (males: β per quantile change = 0.012, 95% CI of 0.002, 0.022 (*p* = 0.020); female: β per quantile change = 0.036; 95% CI of 0.023, 0.048 (*p* < 0.001)). The GAM illustrated that the non-linear associations between blood Mn levels and VAT mass were in U-shape patterns (effective degree of freedom >1 in total participants, males, and females). A stratified analysis found significant interactions between Mn and the family income-to-poverty ratio (PIR) in males, with stronger associations in males with a PIR < 1.3 (β = 0.109; 95% CI of 0.048, 0.170). Additional analyses revealed that individuals in the highest quantile of Mn had a 39% higher risk of visceral obesity (OR = 1.39; 95% CI of 1.15–1.69; *p* < 0.001). Conclusions: Higher blood Mn levels were positively associated with increased VAT mass and visceral obesity risk. The adverse VAT phenotype associated with excessive blood Mn levels should be further investigated.

## 1. Introduction

Obesity in the population tends to be a notable public health threat on the global scale [1]. The prevalence of obesity is increasing rapidly in the US, and individuals with higher body mass index (BMI) or fat mass are considered to have a lower life expectancy [2,3,4]. Among the obesity-related indicators, visceral adipose tissue (VAT) is acknowledged an emerging and sensitive predictor for the risk of metabolic syndrome, cancer, cardiovascular disease, and related outcomes [5,6]. A previous large-scale cohort study also revealed the underlying causality of excess VAT accumulation and diseases [7]. Therefore, adverse VAT accumulation can influence metabolic health in the population, and its related risk factors should be further investigated.

Manganese (Mn) is an essential trace element with a narrow safety margin for human health [8]. According to previous research, moderate Mn intake in the daily diet reduces the oxidative stress level and is associated with a lower health risk [9]. However, excessive Mn in the human body, especially environmental Mn, which come from wide range of sources in the environment (including air, dust, water, soil, industrial pollution, and man-made containments such as battery or ferroalloy production), may cause an assignable health burden [10,11,12]. Previous studies determined that excessive Mn levels may result in cellular toxicity, inflammation, or oxidative stress using population survey or animal experiments [13,14]. Presently, few studies have been conducted to analyze the associations between body Mn levels and metabolic dysfunction. Recently, a study based on a general population survey displayed that blood Mn levels are associated with liver steatosis [15], suggesting that Mn is related to metabolic alterations. Though previous studies revealed the potential effect of Mn on lipid metabolic disorder, the evidence is limited in terms of elucidating the association between Mn levels and the phenotype of adipose accumulation.

Considering the potential metabolic toxicity of overdose Mn levels, we hypothesized that long-term excessive Mn levels could be associated with abnormal fat distribution such as VAT accumulation, while a threshold value might exist in the toxic effect of Mn [13,16,17]. Here, we conducted a population-based study to estimate the associations between blood Mn levels and VAT mass. Our study enrolled 7297 adults from National Health and Nutrition Examination Survey (NHANES) to clarify the relations between blood Mn and VAT measured using dual-energy X-ray absorptiometry (DXA), an advanced technology that can estimate body VAT rapidly and accurately [7]. Using a generalized linear regression model, we estimated the linear associations between blood Mn levels and VAT mass in the general population. The non-linearity of the associations was estimated using the generalized additive model (GAM). Our study can provide insights into a potential link between excessive Mn levels and VAT mass or visceral obesity.

## 2. Methods

### 2.1. Study Population

This cross-sectional study used data collected via personal interviews, physical examinations, and laboratory tests from NHANES (2011–2018). NHANES is a population-based survey using complex, multistage, and probability sampling methods to obtain representative data of the general population in the US. The protocol for NHANES was approved by the National Center for Health Statistics (NCHS) Ethics Review Committee, and detailed information is accessible on their website [18].

**Figure 1:**
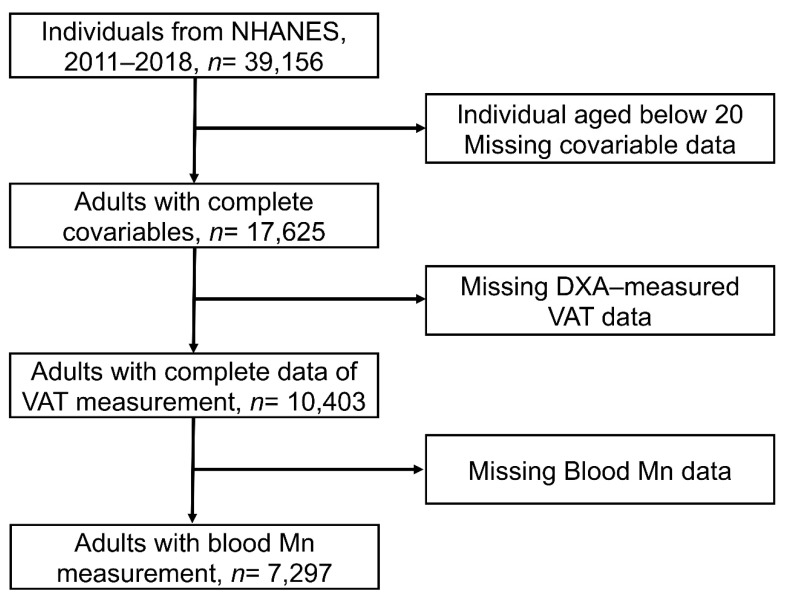
Flow chart of the study.

In this study, only adults aged 20 years or over were included. Participants with missing data about VAT measurements, blood Mn, or other covariables were excluded. The flow chart of our study is shown in Figure 1. Therefore, our research study enrolled 7297 individuals from NHANES. All individuals presented informed consent. All individuals with missing values of exposure, outcomes, and covariables were excluded. The protocol for NHANES passed the review of the NCHS Ethics Review Committee. Data analyses were conducted from October 2021 to January 2022. Our cross-sectional study followed the Strengthening the Reporting of Observational Studies in Epidemiology (STROBE) reporting guidelines.

### 2.2. Exposure Assessments

Generally, in NHANES 2011–2018, whole blood samples were collected and diluted for the measurements. Inductively coupled plasma mass spectrometry with dynamic reaction cell (ICP-DRC-MS) were used for the quantification of Mn [19].

### 2.3. Outcomes

In 2011–2018, individuals aged 8–59 years were DXA-examined at NHANES mobile examination centers. DXA is an advanced and widely used technology that can quantify the body composition with high accuracy and efficiency. VAT was defined using Hologic APEX software in USA in the scan analysis. The mass and area of the intra-abdominal VAT were measured at the approximate interspace between the L4 and L5 vertebrae. Pregnant individuals were excluded. Individuals with a self-reported history of radiographic contrast material (barium) use in the past 7 days and those with a measured weight of over 450 pounds or a height of over 6′5′′ were identified as invalid measurements for the limitations of DXA measurements. Visceral obesity was defined as a VAT area ≥100 cm^2^ [20].

### 2.4. Covariates

Covariates with potential modifying effects on the associations between blood Mn and VAT mass were selected. Generally, age, gender, race, educational level, BMI, marital status, family income-to-poverty ratio (PIR), smoking status, 24 h alcohol consumption, physical activity, 24 h energy intake, and 24 h fat intake were included. Demographic characteristics were collected from self-reported interviews of individuals [21]. Race was categorized in NHANES as Mexican American, Other Hispanic, non-Hispanic White, non-Hispanic Black, non-Hispanic Asian, and other races. The educational level was categorized as less than 9th grade, 9–11th grade, high school graduate/General Educational development (GED) or equivalent, college or Associate of Arts (AA) degree, and college graduate. The marital status was coded as married, separated/divorced/widowed, and never married. The family PIR is a variable calculated by dividing family (or individual) income by the poverty guidelines specific to the survey year (the midpoint of the range is used if the income is reported as a more detailed category). The family PIR was grouped as <1.3, 1.3–3.5, and >3.5 [22]. The BMI was calculated by dividing weight in kilograms by height in square meters [23]. The smoking status (smoker, non-smoker) of individuals was also collected and was determined using the question, “Have you smoked at least 100 cigarettes in entire life?”. Considering the potential effect of dietary factors including alcohol consumption, energy intake, and fat intake on VAT, a 24 h dietary recall was used to obtain related information [24,25]. The dietary nutrient intake in NHANES was calculated following the US Food and Nutrient Database for Dietary (FDNN) database for the US [26]. Alcohol consumption was categorized into 5 levels as 0 g, 0.1 g–4.9 g, 5 g–14.9 g, 15 g–29.9 g, and ≥30 g [22]. Fat and energy intakes were included as continuous variables. Physical activity is acknowledged as a vital factor associated with fat distribution [27]; the intensity of vigorous-to-moderate physical activity time per week (defined as the sum of time individual spent on moderate-to-vigorous recreational physical activity in a typical week) was obtained through the questionnaire and was coded as 0 h/week, >0 h/week–<1 h/week, ≥1 h/week–<3.5 h/week, ≥3.5 h/week–<6 h/week, and ≥6 h/week [22].

### 2.5. Statistical Analysis

Before the analyses, the VAT mass and blood Mn data were all log-nature (ln)-transformed considering their right-skewed distribution [28]. We categorized the blood Mn levels of the included individuals (*n* = 7297) in four quantiles. Unweighted demographic characteristics were displayed to show the distribution of the variables. We used the ANOVA and chi-square methods to test if there were differences in the baseline variables among individuals divided into four Mn quantiles. Student’s *t*-test was performed to compare the differences in VAT mass among the four different quantiles of Mn exposure. Multivariate generalized linear regression was used to estimate the associations between blood Mn levels and VAT mass in total individuals, males, and females [29]. Associations were expressed as coefficients (β) with their 95% confidence interval (CI) to estimate the increment in ln-transformed VAT with per quantile change. We also fitted the ln-transformed blood Mn variable to the models to estimate the trends for associations between blood Mn levels and VAT mass. In minimally adjusted models, age (continuous), gender (male or female, only in total population), and race (Mexican American, other Hispanic, non-Hispanic White, non-Hispanic Black, non-Hispanic Asian, and other race) were fitted as covariates. Fully adjusted models were adjusted for age, gender (only in total individuals), race, educational level (less than 9th grade, 9–11th grade, high school graduate/GED or equivalent, college or AA degree, and college graduate or above), BMI (<25 kg/m^2^, 25–29.9 kg/m^2^, and ≥30 kg/m^2^), marital status (married, separated/divorced or widowed, and never married), PIR (<1.3, 1.3–3.5, and >3.5), smoking status (smoker, non-smoker), 24 h alcohol consumption (0 g, 0.1 g–4.9 g, 5 g–14.9 g, 15 g–29.9 g, and ≥30 g), physical activity (0 h/week, >0 h/week–<1 h/week, ≥1 h/week–<3.5 h/week, ≥3.5 h/week–<6 h/week, and ≥6 h/week), 24 h energy intake (continuous), and 24 h fat intake (continuous).

We also estimated the complex non-linear relationships using the GAM [30]. We established the GAM with Gaussian family and smoothers as cubic regression splines [8]. All models were adjusted for age, gender (only in total populations), race, educational level, BMI, marital status, PIR, smoking status, 24 h alcohol consumption, physical activity, 24 h energy intake, 24 h fat intake. We applied the effective degree of freedom (edf) to estimate non-linearity, where edf = 1 was defined as no non-linearity and edf > 1 was defined as non-linearity [31,32]. All models were adjusted for age, gender (only in total populations), race, educational level, BMI, marital status, PIR, smoking status, 24 h alcohol consumption, physical activity, 24 h energy intake, and 24 h fat intake. Stratified analyses were performed in both males and females as an exploratory procedure to determine interactions between covariables and blood levels. We estimated the potential modifying effects of confounders (age, BMI, and PIR) by calculating the *p* interaction for cross products of Mn exposure and related covariates. We also constructed GAMs in subpopulations to estimate the non-linear association.

Additional analyses were also performed to estimate the association between Mn exposure and the risk of visceral obesity. We set the visceral obesity phenotype as a VAT area ≥100 cm^2^, which was previously defined in published clinical research [20]. The logistic regression model was used to estimate the Mn-associated risks of visceral obesity. The odds ratio (OR) with 95% CI was calculated using exp(beta(95% CI)) and was used to quantify the risks.

In NHANES, weights were used to adjust the selection bias caused by age, gender, and race. However, several studies revealed that the application of weights can cause over-adjustment in models already containing three demographic variables [28]. Therefore, we applied unweighted models in our main analysis for accurate estimation, as performed in previous studies [28,33].

We performed additional sensitivity analyses for validating the results of the main analysis. First, we additionally adjusted 24 h recall protein and carbohydrate consumption into our models. Second, we added high-density lipoprotein (HDL) and total cholesterol (TC) as covariates in our models for the adjustment of metabolic functions. Third, we further adjusted baseline diseases as self-reported cancer, self-reported hypertension, and self-reported diabetes for estimations. Fourth, we adjusted survey weight, strata, and PSU as recommended in NHANES to test the robustness of our results [34]. Fifth, we further adjusted urinary Mn (adjusted with urinary creatinine) to see the effects of metabolism of Mn on the results, in which urinary Mn levels were found to be positively associated with Blood Mn levels (β = 0.046; 95% CI: 0.031, 0.060; *p* < 0.001). To avoid potential bias caused by missing values, we calculated the quantiles from the total included individuals (*n* = 7297) and fitted the quantiles in the models for the sensitivity analyses.

Analyses were performed using R version 3.6.1 in mac OS. The packages *foreign*, *survey 4.1-1*, *mgcv*, and *ggplot2* were used for analysis procedure and visualization. A two-tailed *p*-value < 0.05 was considered statically significant.

## 3. Results

### 3.1. Demographic Characteristics

A total of 39,156 individuals were enrolled in NHANES 2011–2018, and we excluded individuals with missing or invalid values of VAT measurements, covariates, and Mn exposure. Finally, our analysis included 7297 individuals (3703 males and 3594 females). The demographic characteristics of our study populations are displayed in Table 1. Significant differences were observed in age, BMI, 24 h energy intake, 24 h fat intake, gender, race, educational level, marital status, alcohol consumption, and smoking status among individuals in different quantiles.

### 3.2. Blood Mn Levels and VAT Mass

Blood Mn and VAT mass are shown in Table S1. The geometric mean (GM) of blood Mn in the total participants was 9.56 μg/L, while the GM was 8.85 μg/L in males and 10.36 μg/L in females. The GM of VAT mass was 454.13 g in males, higher than the 391.46 g in females.

### 3.3. Associations between Blood Mn Levels and VAT Mass

Figure S1A displays the VAT mass in individuals in different quantiles of blood Mn levels. Significantly higher levels of VAT mass were observed in individuals in quantile 3 or quantile 4 than those in quantile 1. Figure S1B,C display the VAT mass in males and females; similar patterns of VAT mass distribution among quantiles were observed. Figure S1D shows the crude β of associations between blood Mn levels and VAT mass. We found that blood Mn levels were positively associated with VAT mass in the crude model.

Next, we estimated the covariable-adjusted associations between blood Mn exposure and VAT mass. As shown in Table 2, in the fully adjusted model, blood Mn levels were found to be positively associated with VAT mass (β per quantile change = 0.025; 95% CI of 0.017, 0.033; *p* < 0.001). In males and females, the associations also displayed the same patterns (male: β per quantile change = 0.012, 95% CI of 0.002, 0.022 (*p* = 0.020); female: β per quantile change = 0.036, 95% CI of 0.023, 0.048 (*p* < 0.001)). Fitting the ln-transformed Mn into the model, the trends of the positive associations were found to be significant in total, male, and female individuals (*p* for total individuals < 0.001; *p* for females < 0.001; *p* for males = 0.005).

### 3.4. Non-Linear Associations between Blood Mn and VAT Mass

**Figure 2:**
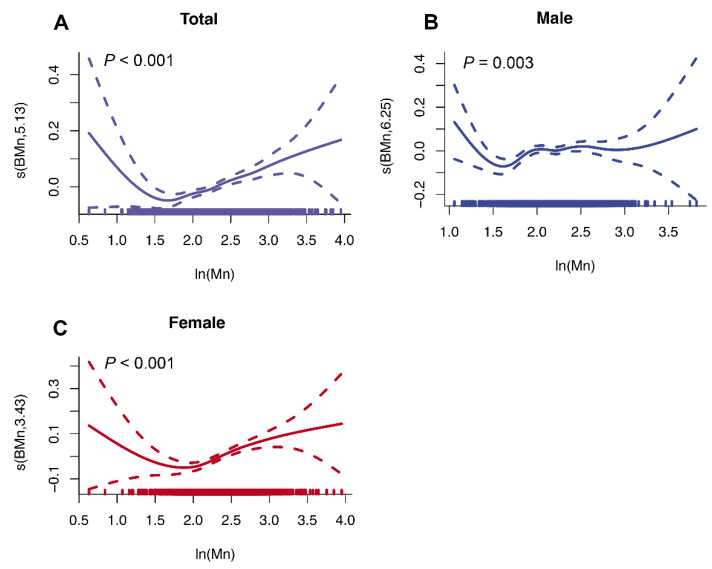
**Non-linear associations between blood Mn levels and VAT mass using generalized additive model (GAM):** (**A**) Dose–response association between blood Mn levels and VAT mass in the total dataset. (**B**) Dose–response association between blood Mn levels and VAT mass in the male subpopulation. (**C**) Dose–response association between blood Mn levels and VAT mass in the female subpopulation. Models were adjusted for gender (only in total), age, race, educational level, BMI, marital status, PIR, smoking status, 24 h alcohol consumption, physical activity, 24 h energy intake, and 24 h fat intake.

The GAM was applied to estimate the dose–response curve of associations between blood Mn exposure and VAT mass. As Figure 2 displays, the non-linear associations between Mn exposure and VAT mass were estimated as U-shape patterns in total (*p* < 0.001, edf = 5.13), male (*p* = 0.003, edf = 6.25), and female (*p* < 0.001, edf = 3.43) individuals, which were found to be non-linear relations. Notably, the value at the turning point for females was higher than that for males, suggesting potential disparities in the Mn–VAT associations among genders.

### 3.5. Sensitivity Analysis and Stratified Analysis

The associations were consistent in the sensitivity analyses (Tables S2–S6). After additional adjustment for HDL and TC, the positive associations were not modified. The association remained positive in the models additionally adjusted for self-reported baseline diseases or protein and carbohydrate consumption in the 24 h dietary recall. Moreover, the application of NHANES-recommended survey weights did not alter the results, determining that the associations were population representative.

**Figure 3:**
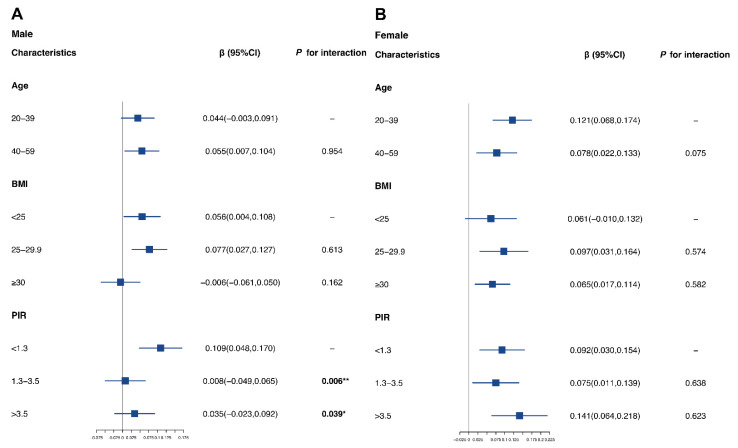
**Stratified analyses on male and female subpopulations:** (**A**) Stratified analysis of Mn and VAT mass in males. (**B**) Stratified analysis of Mn and VAT mass in females. Models were all adjusted for age, race, educational level, BMI, marital status, PIR, smoking status, 24 h alcohol consumption, physical activity, 24 h energy intake, and 24 h fat intake. The *p*-value for cross products of ln-transformed Mn levels and related stratified variables was calculated to estimate the potential interaction. *: *p* value < 0.05; **: *p* value < 0.01.

Subsequent analyses stratified by age, BMI, and PIR were performed in both males and females (Figure 3). We found a stronger association (β = 0.109; 95% CI of 0.048, 0.170) in males with the lowest PIR (PIR < 1.3), while significant interactions were also found (*p* interaction for PIR 1.3–3.5 = 0.006; *p* interaction for PIR >3.5 = 0.039). Regarding BMI-stratified analyses, we found the strongest association in overweight females or males (β for overweight females = 0.097 and 95% CI of 0.031, 0.164; β for overweight males = 0.077 and 95% CI of 0.027, 0.127). We also performed stratified GAM analyses to estimate the non-linear associations in subpopulations (Figures S2–S7). Generally, an obvious heterogeneity of non-linear patterns was found in different subpopulations, indicating potential differences in the relationship between Mn exposure and VAT mass.

### 3.6. Associations between Mn Exposure and Visceral Obesity Risk

**Figure 4:**
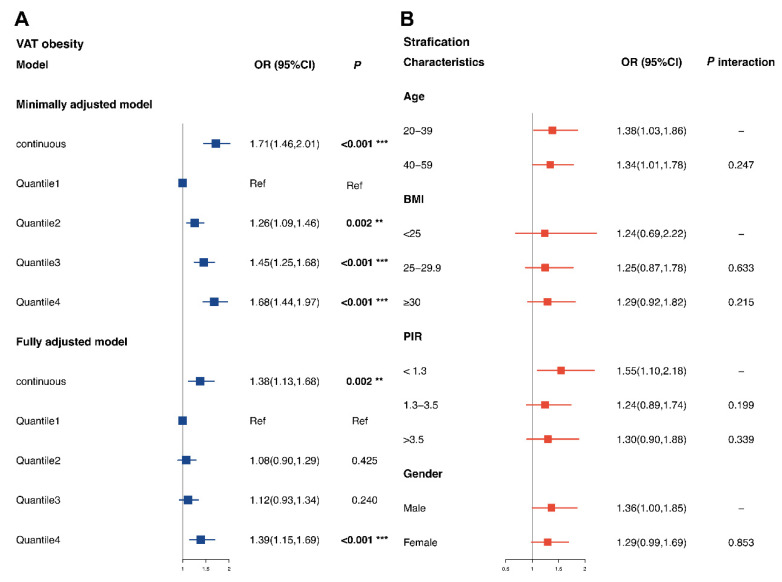
**Associations between blood Mn levels and visceral obesity risk.** (A). Associations between blood Mn levels and visceral obesity in total subjects. Minimally adjusted models were adjusted for gender (only in total), age and race. Fully adjusted models were adjusted for gender, age, race, educational level, BMI, marital status, PIR, smoking status, 24-h alcohol consumption, physical activities, 24-h energy intake, 24-h fat intake. (B). Stratified associations between blood Mn levels and visceral obesity. **: *p* value < 0.01, ***: *p* value < 0.001.

As shown in Figure 4A, in the fully adjusted model, the highest quantile of blood Mn level was associated with a 39% increased risk of visceral obesity than the lowest quantile (OR for Q4 = 1.39; 95% CI of 1.15–1.69; *p* < 0.001). The test for trends also displayed the same patterns for the associations (*p* for trend = 0.002). The results of the stratified analyses are displayed in Figure 4B. We found that the risk of visceral obesity was higher in males, individuals aged 20–39, and individuals with a PIR 1.3 below (OR for males of 1.36 and 95% CI of 1.00–1.85; OR for individuals aged 20–39 of 1.38 and 95% CI of 1.03–1.86; OR for individuals with a PIR <1.3 of 1.55 and 95% CI of 1.10–2.18). No interactions were found between blood Mn exposure and selected variables (age, gender, PIR, and BMI). However, these results should be further validated, because they were estimated in an explorative analysis based on previous evidence for definitions of visceral obesity.

## 4. Discussion

In this cross-sectional study, we estimated the positive associations between blood Mn levels and VAT mass in the general US population. We observed U-shaped associations between blood Mn levels and VAT mass with GAM analyses, while stratified analyses revealed the potential disparities among associations in sub-populations. Additional analyses indicated that the highest quantile of Mn was associated with a 39% increase in visceral obesity risk. Our study highlighted that excessive blood Mn levels were linked to adverse VAT accumulation and could play a vital role in visceral obesity risk.

Previous epidemiological research showed that excessive Mn levels are associated with the increased risk of metabolic disfunction and abdominal obesity. A study using adults’ data from NHANES 2011–2016 exhibited a U-shaped association between urinary Mn and the risk of metabolic syndrome [17]. Another study revealed that blood Mn levels in children are positively associated with waist circumstances (an alternative indicator to characterize visceral obesity) and serve as the main contributors in the co-exposure model [35]. In a study conducted in Poland, consistent associations were also observed [36]. Our study obtained consistent results and provided solid evidence using advanced DXA data of the general population.

The U-shape associations between Mn and VAT mass can be confirmed in experimental studies. On one hand, moderate Mn intake can rescue the phenotypes of metabolic alterations, including obesity, adipose accumulation, or diabetes. On the other hand, excessive Mn levels could increase reactive oxygen species (ROS) generation, thus resulting in oxidative stress, inflammation, and apoptosis [37,38,39], which are highly correlated with adverse adipose accumulation in the human body [40,41]. Excessive Mn storage in hepatocytes upregulates the phosphorylation of JNK and further increases the expression level of key regulators (p53 or c-JUN protein) in the apoptosis pathway [42,43]. Another study revealed that overdose Mn could induce lipid accumulation and lipogenesis in the intestine via the oxidative stress-SIRT1-PPARγ pathway [44]. In mitochondria, Mn may distort the function of the mitochondrial enzyme complex and triggers oxidative stress or inflammation [45,46], resulting in metabolic disfunction associated with VAT mass accumulation. Updated experimental research also found that Mn levels can dysregulate TFEB, Beclin1, Bcl-2, and mTOR proteins and induce autophagy dysregulation [47,48], a potential pathway linked to visceral obesity.

Additionally, we found gender-derived disparities in the associations between blood Mn levels and VAT accumulation. This could be caused by differences in genetics, lifestyle, and Mn absorption patterns between genders [8]. Compared with males, females had stronger associations and had a higher turning point value in the non-linear associations. An experimental study based on C57BL/6 mice also indicated that Mn exposure could disrupt the pro-inflammatory mediators, including gut microbiome or metabolites, the effects of which were stronger in female mice [49]. This finding could partly explain our results. Additionally, in males, significant interactions were found between the PIR and blood Mn levels, and a specific, significant association was observed in males with lower income. The modification caused by the PIR in the associations may arise from the heterogeneity of lifestyles, access to health care, living environment, or dietary quality among males with different socio-economic statuses.

Our study further revealed that higher blood Mn levels are a risk factor for clinical visceral obesity (defined using DXA technology). Visceral obesity was reported to be highly associated with several non-communicable diseases (NCDs), including cardiovascular disease, diabetes, and cancer [7,50,51]. Therefore, the metabolic health and life expectancy loss attributable to excessive Mn exposure should be evaluated in the future.

Our study had several strengths. First, we applied VAT data measured using DXA technology, which could quantify visceral adipose tissue more accurately than traditional indicators, such as waist circumference or BMI [52]. Second, because of the multi-stage design of NHANES, our enrolled subjects were population representative, and we had a large sample size, which could provide reliable associations between blood Mn levels and VAT mass in the general US population [18]. Third, with the application of the GAM, the dose–response associations between blood Mn levels and VAT mass could be better estimated [30]. Therefore, our study implies that excess blood Mn levels are associated with VAT accumulations in adults, pointing at the practical importance of controlling for excessive Mn exposure in the population, as it would be helpful in reducing VAT accumulation and the related burden of disease in both males and females.

The study had several limitations. First, the blood Mn levels at the selected time points may be insufficient to reflect exposure over the long term. Second, blood Mn analysis can barely distinguish between industrial exposure and dietary exposure. According to previous studies, Mn in industrial or other pollutants has different effects on the human body compared with dietary Mn [42,53]. Therefore, we recommend that experimental studies are established in the future to clarify the influence of industrial Mn or dietary Mn on human VAT accumulation. Third, because of the nature of the cross-sectional study, the results are limited in verifying the causality of Mn exposure on VAT mass or visceral obesity risk [54], as several unmeasured health confounding factors, including the living environment, metabolites, and genetic structures, may also affect our findings [7,55,56]. Therefore, cohort studies or causality inference methods such as mendelian randomization should be conducted to validate our results. Fourth, our study only included young and middle-aged individuals (individuals aged 20–59), while individuals aged 60 years or over were not included in our study. Future studies on blood Mn levels and VAT in the elders should be performed to expand the findings.

## 5. Conclusions

Higher blood Mn levels were positively associated with an increased VAT mass or visceral obesity risk. Our study points at the practical importance of controlling for excessive Mn exposure in the population, as it would be helpful in reducing VAT accumulation and the related burden of disease in both males and females.

## Figures and Tables

**Table 1:** Unweighted demographic characteristics of our study population (*n* = 7297).

Characteristic ^a^	Quantile of Blood Mn Levels				*p*-Value ^b^
	Quantile 1 (1.88–7.59) *n* = 1827	Quantile 2 (7.60–9.44) *n* = 1823	Quantile 3 (9.45–11.86) *n* = 1823	Quantile 4 (11.87–52.00) *n* = 1824	
**Age (mean (SD)), year**	40.20 (11.60)	39.25 (11.89)	39.54 (11.61)	38.78 (11.00)	0.002
**BMI (mean (SD)), kg/m^2^**	28.68 (6.66)	28.98 (6.78)	29.55 (7.10)	29.13 (7.28)	0.002
**PIR (mean (SD))**	2.54 (1.66)	2.55 (1.67)	2.50 (1.65)	2.49 (1.67)	0.624
**24 h energy intake (mean (SD)), kcal**	2364.94 (1100.27)	2308.76 (1065.27)	2223.33 (1010.60)	2125.35 (950.04)	<0.001
**24 h fat intake (mean (SD)), g**	91.92 (52.34)	87.85 (50.36)	85.58 (47.83)	80.49 (45.05)	<0.001
**Gender**					
Male	1161 (63.5)	1019 (55.9)	896 (49.1)	627 (34.4)	<0.001
Female	666 (36.5)	804 (44.1)	927 (50.9)	1197 (65.6)	
**Race**					
Mexican American	164 (9.0)	198 (10.9)	266 (14.6)	332 (18.2)	<0.001
Other Hispanic	144 (7.9)	181 (9.9)	182 (10.0)	182 (10.0)	
Non-Hispanic White	737 (40.3)	744 (40.8)	704 (38.6)	509 (27.9)	
Non-Hispanic Black	640 (35.0)	451 (24.7)	308 (16.9)	223 (12.2)	
Non-Hispanic Asian	59 (3.2)	153 (8.4)	284 (15.6)	501 (27.5)	
Other race—including multi-racial	83 (4.5)	96 (5.3)	79 (4.3)	77 (4.2)	
**Educational level**					
Less than 9th grade	76 (4.2)	93 (5.1)	89 (4.9)	124 (6.8)	<0.001
9–11th grade (includes 12th grade with no diploma)	199 (10.9)	199 (10.9)	228 (12.5)	205 (11.2)	
High school graduate/GED or equivalent	447 (24.5)	397 (21.8)	375 (20.6)	366 (20.1)	
College or AA degree	657 (36.0)	637 (34.9)	594 (32.6)	556 (30.5)	
College graduate or above	448 (24.5)	497 (27.3)	537 (29.5)	573 (31.4)	
**BMI category, kg/m^2^**					
<25	573 (31.4)	551 (30.2)	531 (29.1)	578 (31.7)	0.046
25–29.9	608 (33.3)	563 (30.9)	552 (30.3)	553 (30.3)	
≥30	646 (35.4)	709 (38.9)	740 (40.6)	693 (38.0)	
**Marital status**					
Married	810 (44.3)	822 (45.1)	893 (49.0)	933 (51.2)	<0.001
Separated, divorced, or widowed	286 (15.7)	256 (14.0)	268 (14.7)	245 (13.4)	
Never married	731 (40.0)	745 (40.9)	662 (36.3)	646 (35.4)	
**PIR**					
<1.3	579 (31.7)	589 (32.3)	588 (32.3)	614 (33.7)	0.845
1.3–3.5	670 (36.7)	649 (35.6)	669 (36.7)	636 (34.9)	
>3.5	578 (31.6)	585 (32.1)	566 (31.0)	574 (31.5)	
**24 h alcohol consumption, g**					
0	1248 (68.3)	1301 (71.4)	1370 (75.2)	1484 (81.4)	<0.001
0.1–4.9	16 (0.9)	14 (0.8)	10 (0.5)	19 (1.0)	
5–14.9	96 (5.3)	95 (5.2)	86 (4.7)	50 (2.7)	
15–29.9	131 (7.2)	136 (7.5)	105 (5.8)	109 (6.0)	
≥30	336 (18.4)	277 (15.2)	252 (13.8)	162 (8.9)	
**Smoking status**					
Smoker	824 (45.1)	799 (43.8)	683 (37.5)	608 (33.3)	<0.001
Non-smoker	1003 (54.9)	1024 (56.2)	1140 (62.5)	1216 (66.7)	
**Physical activity, h/week**					
0	779 (42.6)	809 (44.4)	802 (44.0)	869 (47.6)	0.065
>0–<1	63 (3.4)	60 (3.3)	67 (3.7)	62 (3.4)	
≥1–<3.5	428 (23.4)	416 (22.8)	451 (24.7)	410 (22.5)	
≥3.5–<6	225 (12.3)	208 (11.4)	228 (12.5)	208 (11.4)	
≥6	332 (18.2)	330 (18.1)	275 (15.1)	275 (15.1)	

^a^: The data were unweighted. The bold is represent the main variables. ^b^: ANOVA and chi-square methods were used to test if there were differences in the baseline variables among individuals in each Mn quantile.

**Table 2:** Associations of blood Mn levels and male VAT mass in the US general population.

Characteristics	Minimally Adjusted Model ^a^		Fully Adjusted Model ^b^	
	β (95% CI)	*p*-Value	β (95% CI)	*p*-Value
**β per ln-unit change**				
Total	0.181 (0.144, 0.218)	<0.001	0.086 (0.059, 0.112)	<0.001
Male	0.102 (0.056, 0.148)	<0.001	0.049 (0.015, 0.082)	0.005
Female	0.239 (0.183, 0.295)	<0.001	0.103 (0.064, 0.141)	<0.001
**β per quantile change**				
Total	0.055 (0.043, 0.066)	<0.001	0.025 (0.017, 0.033)	<0.001
Male	0.032 (0.018, 0.045)	<0.001	0.012 (0.002, 0.022)	0.020
Female	0.076 (0.057, 0.094)	<0.001	0.036 (0.023, 0.048)	<0.001

^a^: Minimally adjusted models were adjusted for gender (only in total), age, and race. ^b^: Fully adjusted models were adjusted for gender (only in total), age, race, educational level, BMI, marital status, PIR, smoking status, 24 h alcohol consumption, physical activity, 24 h energy intake, and 24 h fat intake.

## Data Availability

The datasets used in the current study are available on the NHANES website: https://www.cdc.gov/nchs/nhanes/ (accessed on 2 April 2022).

## Supplementary Materials

The following supporting information can be downloaded at: https://www.mdpi.com/article/10.3390/nu14224770/s1, Figure S1. Characterization for blood Mn levels and VAT mass in our study populations. Figure S2. Age-specific dose-response association between Mn exposure and VAT mass in males. Figure S3. BMI-specific dose-response association between Mn exposure and VAT mass in males. Figure S4. PIR-specific dose-response association between Mn exposure and VAT mass in males. Figure S5. Age-specific dose-response association between Mn exposure and VAT mass in females. Figure S6. BMI-specific dose-response association between Mn exposure and VAT mass in females. Figure S7. PIR-specific dose-response association between Mn exposure and VAT mass in females. Table S1. Unweighted concentration of blood Mn in enrolled participants. Table S2. Sensitive analysis I for associations between manganese exposure and VAT mass. Table S3. Sensitive analysis II for associations between manganese exposure and VAT mass. Table S4. Sensitive analysis III for associations between manganese exposure and VAT mass. Table S5. Sensitive analysis IV for associations between manganese exposure and VAT mass. Table S6. Sensitive analysis V for associations between manganese exposure and VAT mass.

## Abbreviations

***AA***	Associate of Arts
***GED***	General Educational development
***NCHS***	National Center for Health Statistics
***BMI***	Body mass index
***VAT***	Visceral adipose tissue
***Mn***	Manganese
***NHANES***	National Health and Nutrition Examination Survey
***DXA***	Dual-energy X-ray absorptiometry
***GAM***	Generalized additive model
***STROBE***	Strengthening the Reporting of Observational Studies in Epidemiology
***ICP-DRC-MS***	Inductively coupled plasma mass spectrometry with dynamic reaction cell
***PIR***	Family income-to-poverty ratio
***OR***	Odds ratio
