# Supplementary material for: Association between Blood Manganese Levels and Visceral Adipose Tissue in the United States: A Population-Based Study
Source: Nutrients. 2022 Nov 11;14(22):4770. doi: 10.3390/nu14224770 (PMC9697925; doi:10.3390/nu14224770)
Supplement: Supplementary file 1 [file nutrients-14-04770-s001.zip › nutrients-1973799-supplementary.pdf]

Supplementary materials

Figure S1. Characterization for blood Mn levels and VAT mass in our study populations.
Figure S2. Age-specific dose-response association between Mn exposure and VAT mass in males.
Figure S3. BMI-specific dose-response association between Mn exposure and VAT mass in males.
Figure S4. PIR-specific dose-response association between Mn exposure and VAT mass in males.
Figure S5. Age-specific dose-response association between Mn exposure and VAT mass in females.
Figure S6. BMI-specific dose-response association between Mn exposure and VAT mass in females.
Figure S7. PIR-specific dose-response association between Mn exposure and VAT mass in females.

Table S1. Unweighted concentration of blood Mn in enrolled participants.
Table S2. Sensitive analysis I for associations between manganese exposure and VAT mass.
Table S3. Sensitive analysis II for associations between manganese exposure and VAT mass.
Table S4. Sensitive analysis III for associations between manganese exposure and VAT mass.
Table S5. Sensitive analysis IV for associations between manganese exposure and VAT mass.
Table S6. Sensitive analysis V for associations between manganese exposure and VAT mass.

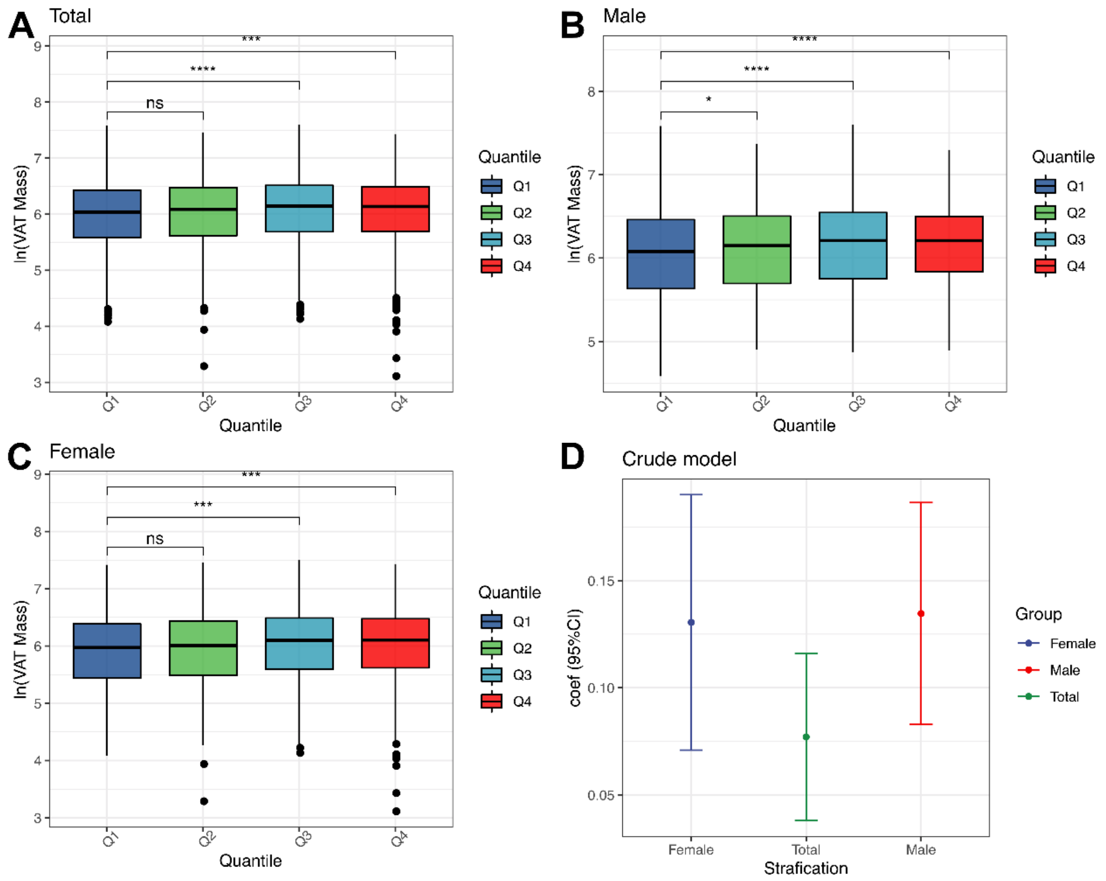

Figure S1. Characterization for blood Mn levels and VAT mass in our study populations. A. VAT mass in different quantiles of blood Mn levels in total population. **B.** VAT mass in different quantiles of blood Mn levels in males. **C.** VAT mass in different quantiles of blood Mn levels in females. **D.** Crude association between Mn levels and VAT mass in total populations, males and females. Students' *t* test was used to identify the differences of VAT mass among individuals with different quantiles of Mn levels. ns: no significant; *: $P < 0.05$; ***: $P < 0.001$; ****: $P < 0.0001$; ns, not significant.

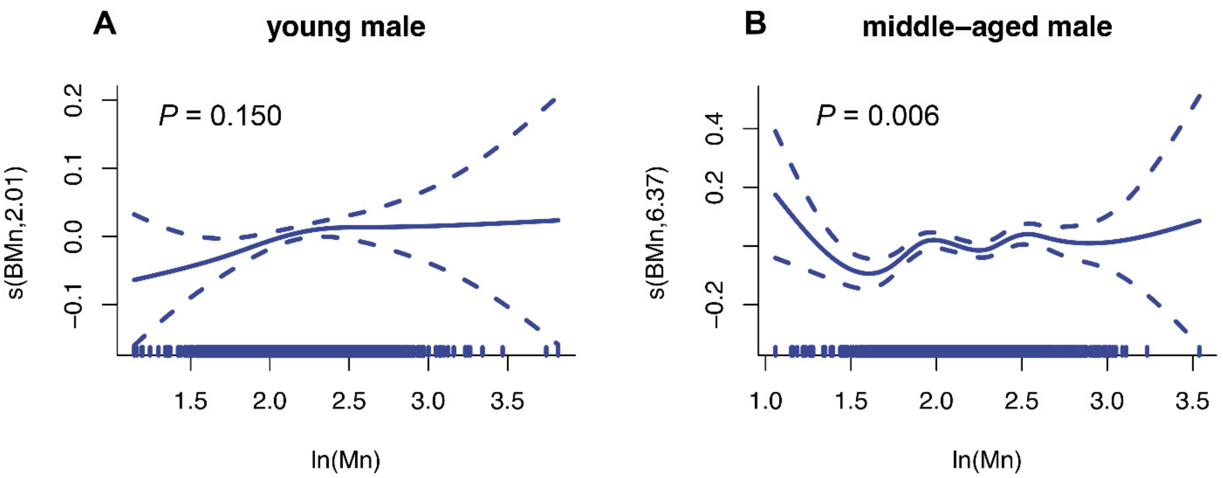

Figure S2. Age-specific dose-response association between Mn exposure and VAT mass in males. A. non-linear associations between blood Mn levels and VAT mass in young males. **B.** non-linear associations between blood Mn levels and VAT mass in middle-aged males. GAM was applied to estimate the non-linear association using the cubic spline function. Models were adjusted for age (continuous), race, educational level, BMI, marital status, PIR, smoking status, 24-hour alcohol consumption, physical activities, 24-hour energy intake, 24-hour fat intake. Age categories were stratified into two groups as young (20-39) and middle-aged (40-59).

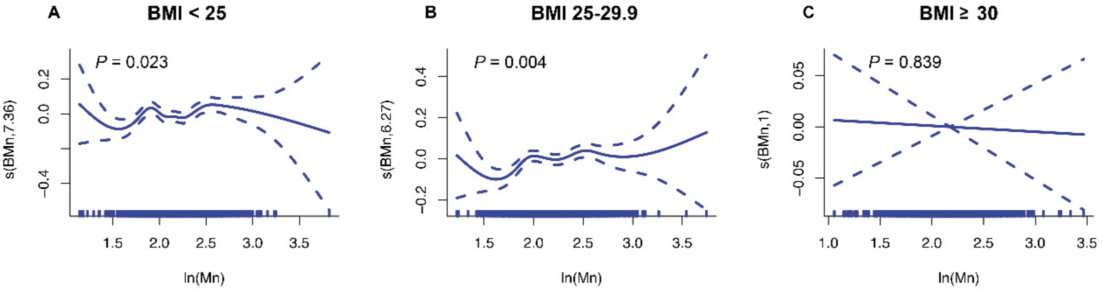

Figure S3. BMI-specific dose-response association between Mn exposure and VAT mass in males. A. non-linear associations between blood Mn levels and VAT mass in males with normal BMI. **B.** non-linear associations between blood Mn levels and VAT mass in overweight males. **C.** non-linear associations between blood Mn levels and VAT mass in obese males. GAM was applied to estimate the non-linear association using the cubic spline function. Models were adjusted for age, race, educational level, BMI (continuous), marital status, PIR, smoking status, 24-hour alcohol consumption, physical activities, 24-hour energy intake, 24-hour fat intake.

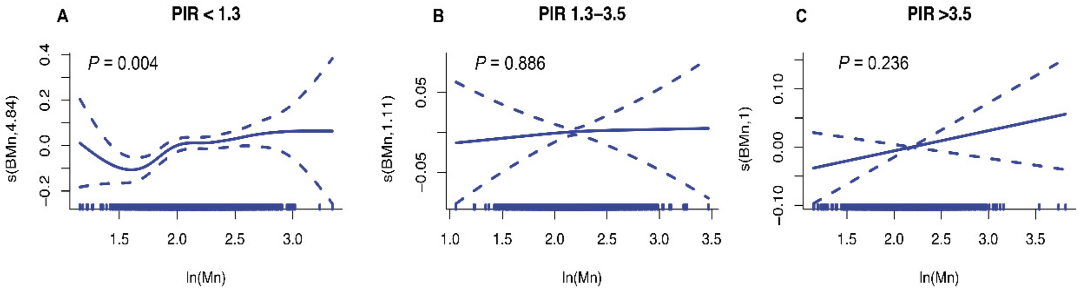

Figure S4. PIR-specific dose-response association between Mn exposure and VAT mass in males. A. non-linear associations between blood Mn levels and VAT mass in males with PIR < 1.3. **B.** non-linear associations between blood Mn levels and VAT mass in males with PIR 1.3-3.5. **C.** non-linear associations between blood Mn levels and VAT mass in males with PIR >3.5. GAM was applied to estimate the non-linear association using the cubic spline function. Models were adjusted for age, race, educational level, BMI, marital status, PIR (continuous), smoking status, 24-hour alcohol consumption, physical activities, 24-hour energy intake, 24-hour fat intake.

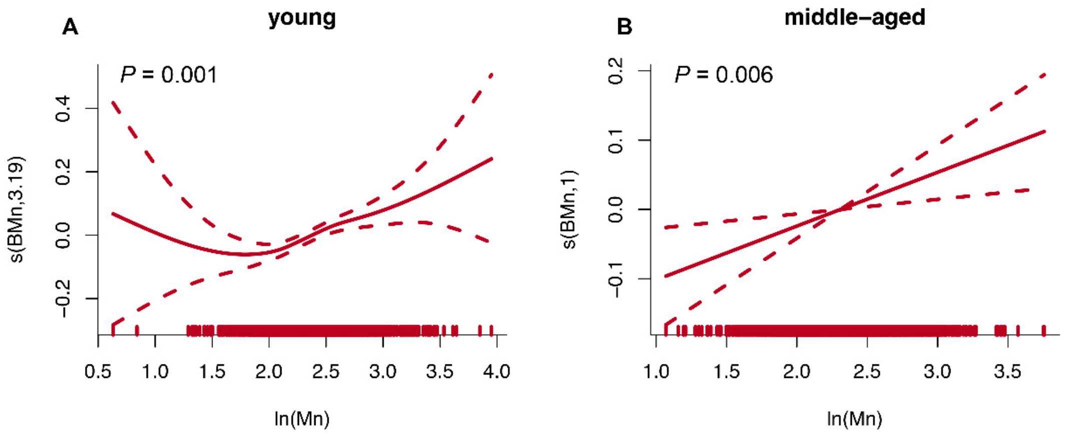

Figure S5. Age-specific dose-response association between Mn exposure and VAT mass in females. A. non-linear associations between blood Mn levels and VAT mass in young females. **B.** non-linear associations between blood Mn levels and VAT mass in middle-aged females. GAM was applied to estimate the non-linear association using the cubic spline function. Models were adjusted for age (continuous), race, educational level, BMI, marital status, PIR, smoking status, 24-hour alcohol consumption, physical activities, 24-hour energy intake, 24-hour fat intake. Age category were stratified into two groups as young (20-39) and middle-aged (40-59).

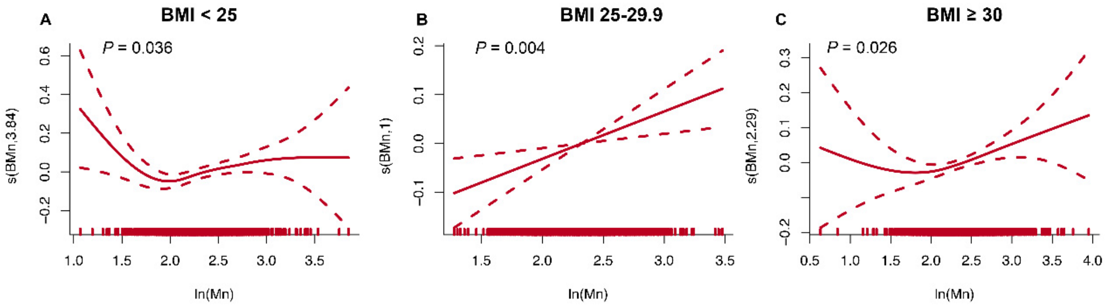

Figure S6. BMI-specific dose-response association between Mn exposure and VAT mass in females. A. non-linear associations between blood Mn levels and VAT mass in females with normal BMI. **B.** non-linear associations between blood Mn levels and VAT mass in overweight females. **C.** non-linear associations between blood Mn levels and VAT mass in obese females. GAM was applied to estimate the non-linear association using the cubic spline function. Models were adjusted for age, race, educational level, BMI (continuous), marital status, PIR, smoking status, 24-hour alcohol consumption, physical activities, 24-hour energy intake, 24-hour fat intake.

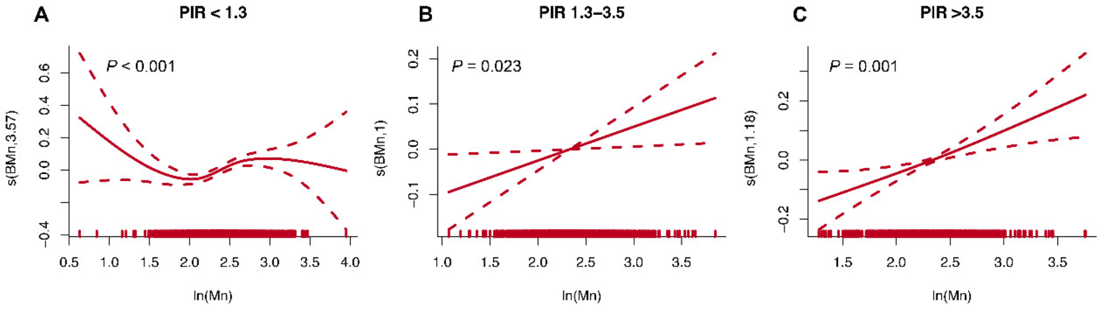

Figure S7. PIR-specific dose-response association between Mn exposure and VAT mass in females. A. non-linear associations between blood Mn levels and VAT mass in females with PIR < 1.3. **B.** non-linear associations between blood Mn levels and VAT mass in females with PIR 1.3-3.5. **C.** non-linear associations between blood Mn levels and VAT mass in females with PIR >3.5. GAM was applied to estimate the non-linear association using the cubic spline function. Models were adjusted for age, race, educational level, BMI, marital status, PIR (continuous), smoking status, 24-hour alcohol consumption, physical activities, 24-hour energy intake, 24-hour fat intake.

Table S1. Unweighted concentration of blood Mn in enrolled participants.

Characteristics [a]	Geometric Mean	Mean	0.05	0.25	0.5	0.75	0.95
**Blood Mn (μg/L)**							
Total	9.56	10.18	5.55	7.59	9.44	11.86	17.21
Male	8.85	9.32	5.38	7.16	8.84	10.84	14.95
Female	10.36	11.06	5.95	8.18	10.22	12.96	18.81
**VAT mass (g)**							
Total	422.10	494.96	146.76	283.76	450.74	653.08	998.92
Male	454.13	515.81	196.84	301.42	469.46	668.10	1008.97
Female	391.46	473.47	116.37	257.59	427.77	635.27	990.25

[a]: Data were unweighted.

Table S2. Sensitive analysis I for associations between manganese exposure and VAT mass.

Characteristics [a]	Fully Adjusted Model	
	**β (95%CI)**	***P* Value**
**β with per ln-unit change**		
Total	0.086 (0.060,0.112)	<0.001
Male	0.050 (0.016,0.083)	0.004
Female	0.104 (0.065,0.142)	<0.001
**β with per quantile change**		
Total	0.025 (0.017, 0.033)	<0.001
Male	0.012 (0.002, 0.022)	0.016
Female	0.036 (0.024, 0.048)	<0.001

[a]: models were adjusted for gender (only in total subjects), age, race, educational level, BMI, marital status, PIR, smoking status, 24-hour alcohol consumption, physical activities, 24-hour energy intake, 24-hour fat intake, 24-hour protein intake and 24-hour carbohydrate intake.

Table S3. Sensitive analysis II for associations between manganese exposure and VAT mass.

Characteristics [a]	Fully Adjusted Model	
	**β (95%CI)**	***P* Value**
**β per ln-unit change**		
Total	0.077 (0.052,0.103)	<0.001
Male	0.045 (0.012,0.078)	0.007
Female	0.091 (0.054,0.129)	<0.001
**β per quantile change**		
Total	0.024 (0.016, 0.032)	<0.001
Male	0.012 (0.002, 0.021)	0.018
Female	0.033 (0.021, 0.045)	<0.001

[a]: models were adjusted for gender (only in total subjects), age, race, educational level, BMI, marital status, PIR, smoking status, 24-hour alcohol consumption, physical activities, 24-hour energy intake, 24-hour fat intake, HDL level and TC level.

Table S4. Sensitive analysis III for associations between manganese exposure and VAT mass.

Characteristics [a]	Fully Adjusted Model	
	**β (95%CI)**	***P* Value**
**β per ln-unit change**		
Total	0.087 (0.061,0.113)	<0.001
Male	0.048 (0.015,0.082)	0.005
Female	0.106 (0.067,0.144)	<0.001
**β per quantile change**		
Total	0.025 (0.017, 0.033)	<0.001
Male	0.013 (0.001, 0.021)	0.024
Female	0.037 (0.024, 0.049)	<0.001

[a]: models were adjusted for gender (only in total subjects), age, race, educational level, BMI, marital status, PIR, smoking status, 24-hour alcohol consumption, physical activities, 24-hour energy intake, 24-hour fat intake, self-reported diabetes, self-reported cancer and self-reported hypertension.

Table S5. Sensitive analysis IV for associations between manganese exposure and VAT mass.

Characteristics [a]	Minimally Adjusted Model [b]		Fully Adjusted Model [c]	
	β (95%CI)	*P* Value	β (95%CI)	*P* Value
**β per ln-unit change**				
Total	0.191 (0.136,0.246)	<0.001	0.095 (0.052,0.137)	<0.001
Male	0.100 (0.040,0.159)	0.002	0.061 (0.014,0.107)	0.012
Female	0.266 (0.182,0.351)	<0.001	0.113 (0.051,0.174)	<0.001
**β per quantile change**				
Total	0.055 (0.039, 0.071)	<0.001	0.025 (0.012, 0.037)	<0.001
Male	0.029 (0.012, 0.046)	0.001	0.012 (-0.003, 0.026)	0.107
Female	0.081 (0.055, 0.108)	<0.001	0.036 (0.017, 0.055)	<0.001

[a]: Survey sampling weights, PSU and strata were applied in the models.

b: Minimally adjusted models were adjusted for gender (only in total), age and race.

c: Fully adjusted models were adjusted for gender (only in total), age, race, educational level, BMI, marital status, PIR, smoking status, 24-hour alcohol consumption, physical activities, 24-hour energy intake, 24-hour fat intake.

Table S6. Sensitive analysis V for associations between manganese exposure and VAT mass.

Characteristics	Fully Adjusted Model [a]	
	β (95%CI)	*P* Value
**β per ln-unit change**		
Total	0.079 (0.039,0.118)	<0.001
Male	0.059 (0.009,0.110)	0.020
Female	0.085 (0.026,0.144)	0.005
**β per quantile change**		
Total	0.022 (0.010, 0.034)	<0.001
Male	0.016 (0.002, 0.031)	0.027
Female	0.026 (0.007, 0.045)	0.007

[a]: Fully adjusted models were adjusted for gender (only in total), age, race, educational level, BMI, marital status, PIR, smoking status, 24-hour alcohol consumption, physical activities, 24-hour energy intake, 24-hour fat intake, and urinary Mn levels.
